# *FTO*-induced *APOE* promotes the malignant progression of pancreatic neuroendocrine neoplasms through *FASN*-mediated lipid metabolism

**DOI:** 10.7150/ijbs.103428

**Published:** 2025-01-27

**Authors:** Jinhao Chen, Mujie Ye, Danyang Gu, Ping Yu, Lin Xu, Bingyang Xue, Lijun Yan, Feiyu Lu, Chunhua Hu, Yanling Xu, Xiaoting Shi, Lingyi Chen, Yan Wang, Jianan Bai, Ye Tian, Qiyun Tang

**Affiliations:** 1 Department of Geriatric Gastroenterology, Neuroendocrine Tumor Center, Jiangsu Province Hospital, The First Affiliated of Nanjing Medical University, Institute of Neuroendocrine Tumor, Nanjing Medical University, Nanjing, China.; 2 Center for Biomarker Discovery and Validation, National Infrastructures for Translational Medicine (PUMCH), Institute of Clinical Medicine, Peking Union Medical College Hospital, Chinese Academy of Medical Science & Peking Union Medical College, Beijing, China.; 3 Digestive Endoscopy, Jiangsu Province Hospital, The First Affiliated Hospital of Nanjing Medical University, China.; 4 Department of Gastroenterology, The Friendship Hospital of Ili Kazakh Autonomous Prefecture, Ili State 835000, China.

**Keywords:** pancreatic neuroendocrine neoplasms, N6-methyladenosine(m6A), *FTO*, *APOE*, lipid metabolism

## Abstract

N6-methyladenosine (m6A) is considered the most prevalent RNA epigenetic regulator in cancer. FTO, an m6A demethylase, has been implicated in contributing to the progression of various cancers by up-regulating the expression of multiple oncogenes. However, studies exploring its impact on lipid metabolism in cancer, especially in pNENs, remain scarce. In this study, we demonstrated that FTO was up-regulated in pNENs and played a critical role in tumor growth and lipid metabolism. Mechanistically, we discovered that FTO over-expression increased the expression of APOE in an m6A-IGF2BP2-dependent manner, leading to dysregulation of lipid metabolism. Furthermore, we found APOE could activate the PI3K/AKT/mTOR signaling pathway, thereby enhancing lipid metabolism and proliferative capabilities, by orchestrating the state of FASN ubiquitination. In conclusion, our study reveals the FTO/IGF2BP2/APOE/FASN/mTOR axis as a mechanism underlying aberrant m6A modification in lipid metabolism and provides new insights into the molecular basis for developing therapeutic strategies for pNENs treatment.

## Introduction

Neuroendocrine neoplasms (NENs) are a heterogeneous group of tumors originating from neuroendocrine cells, occurring in various organs, including the lungs, digestive tract, and pancreas 1. Among gastroenteropancreatic neuroendocrine neoplasms in Asian countries, pNENs are the most common type, accounting for 34.9-38.6% of cases and with an incidence rate of 1.01 per 100,000 individuals 2,3. Although pNENs constitute only 2-5% of all pancreatic cancers, their annual incidence (> 6%) is increasing more rapidly than any other histological subtype 4. Surgery remains the primary and potentially curative treatment for pNENs 5. However, high recurrence rates, up to 48%, have been reported following curative resection 6. The five-year survival rate post-surgery ranges between 65 and 86% 7,8. The management of pNENs involves a multidisciplinary approach, including surgical intervention, somatostatin analogs, molecularly targeted therapies, peptide receptor radionuclide therapy, and cytotoxic chemotherapy 9. Over the years, although the significance of multidisciplinary treatments has grown for effectively controlling tumor development, the availability of molecularly targeted therapies specifically designed for pNENs is currently limited compared to other cancer types, highlighting the need for novel therapeutic targets.

Cellular proliferation is a hallmark of pNENs and cancer in general, requiring enhanced macromolecular biosynthesis to meet structural and energy demands 10. Tumor cells also adapt to environment stressors, such as hypoxia and reactive oxygen species, through extensive metabolic reprogramming 11. While the Warburg effect, increased fatty acid uptake, and elevated *de novo* lipid synthesis are well-documented in other cancers 12, the metabolic remodeling underlying pNENs progression, particularly in lipid metabolism, remains largely unexplored.

Epigenetic modifications, including DNA methylation, histone acetylation, and m6A RNA modification, play crucial roles in tumor metabolic reprogramming 13. N6-methyladenosine (m6A) is the most prevalent internal RNA modification 14, dynamically regulated by the methyltransferases “writers”, demethylases “erasers”, and “readers”. For instance, the key components of the methyltransferase complex, Methyltransferase-like 3 (*METTL3*), Methyltransferase-like 14 (*METTL14*), and Wilms' tumor 1-associated protein (*WTAP*), are responsible for m6A installation 15. Conversely, Fat mass and obesity associated protein (*FTO*) 16 and α-ketoglutarate-dependent dioxygenase alkB homolog 5 (*ALKBH5*) 17 function as erasers, removing m6A removal to maintain a dynamic balance. Additionally, readers, such as *YTHDF1-3* 18, *YTHDC1* 19, Insulin-like growth factor 2 mRNA-binding proteins (*IGF2BPs*) 20, and the heterogeneous nuclear ribonucleoprotein (*HNRP*) protein family 21, recognize and interpret m6A modifications. Not only amounting studies have underscored the essential role of m6A modification in different diseases, the interaction between m6A and remodeling of metabolic has also been reported. For instance, in colorectal cancer, *METTL3/LDHA* axis-induced glucose metabolism could overcome 5-FU resistance to promote tumor development 22. In renal cell carcinoma, downregulated *METTL14* promoted distal lung metastasis via glycolytic reprogramming 23. Additionally, *LINC00958* regulated by m6A modification increased lipogenesis in hepatocellular carcinoma 24. Despite the importance of lipid metabolism and its relevance to different diseases, limited research has been reported on the metabolic alterations of pNENs, particularly concerning the specific metabolism between m6A modification and lipid-related pathways.

In this study, we observed elevated *FTO* expression and its oncogenic effects in pNENs, both *in vitro* and *in vivo*. We further demonstrated that *FTO* enhanced lipid metabolism by regulating the expression of *APOE* in an m6A-*IGF2BP2* dependent manner. Additionally, we identified *FASN* as a critical regulator of lipid metabolism in pNENs, modulated via *APOE* in a ubiquitin-dependent manner. These findings elucidate the role of the *FTO*/*IGF2BP2*/*APOE*/*FASN* axis in pNENs progression and provide a foundation for future therapeutic strategies.

## Methods

### Cell culture and tissue samples

The QGP-1 cell line, derived from human pancreatic neuroendocrine tumors (pNENs), was obtained from the JCRB cell bank (JCRB0183). The cells were cultured in RPMI 1640 medium supplemented with 10% fetal bovine serum (FBS, Yeasen, Shanghai, China) and 1% penicillin-streptomycin. The BON-1 cell line was gift from Professor Xianrui Yu of Fudan University Shanghai Cancer Center, was cultured in DMEM/F-12 medium supplemented with 10% fetal bovine serum and 1% penicillin-streptomycin. The primary PNET cells, cultured in McCoy's 5A medium, was isolated from patient tissue samples. All cells were incubated in a humidified atmosphere with 5% CO_2_ at 37 °C. The pNENs and adjacent normal tissues, diagnosed as pNENs by the Pathology Department, were obtained from Jiangsu Province Hospital. All participants provided their consent.

### Cell proliferation assays

Cell Count-ing Kit-8 (CCK-8, New Cell & Molecular Biotech), colony formation, and EdU assays were conducted to elevate the proliferation abilities. For the CCK-8 assay, different groups of cells (5000 per well) were seeded in a 96-well plate with 5 replicates and then incubated at 37 °C for 2 h. The absorbance at 450 nm was continuously measured for 4 days. For colony formation assays, different groups of cells (1.0x10^4^ per well) were seeded in a 6-well plate for 14 days. The cells were fixed with 4% paraformaldehyde and stained with crystal violet solution. Each experiment was conducted in triplicate. For the 5-Ethynyl-2'-deoxyuridine (EdU) cell proliferation assay, cells were seeded into 96-well plates with 50 μM EdU for 2h, fixed with 4% paraformaldehyde, permeated with 0.3% Triton X-100, and then stained according to the instructions provided (RiboBio, Guangzhou, China).

### Cell migration and invasion assays

Transwell assays were performed in 24-well cell culture plates with 8-μm micropore inserts to examine the ability of migration and invasion. To determine cell migration, the upper chamber was filled with a serum-free medium with 1×10^5^ cells, while the lower chamber was filled with a conditioned culture medium containing 30% FBS. To determine cell invasion, 2 × 10^5^ cells with a serum-free medium were seeded in the upper chamber with 50 μL of Matrigel, while the lower chamber was filled with the same conditioned culture medium. After incubation for 48 h, the invaded cells at the membranes were fixed with 4% paraformaldehyde and stained with 0.5% crystal violet solution.

### Lentivirus transfection and stable cell line construction

*FTO, APOE, FASN*, and *IGF2BPs* knockdowns, as well as over-expression plasmids, were synthesized by Shanghai Genomeditech Biotech Co. Ltd. Lentivirus packaging was conducted in 293 T cells using PEIMAX treatment (Polysciences, USA). Following infection with concentrated virus and 5 μg/ml polybrene (Genomeditech) for 48 hours, stable transfected cells were selected through treatment with 5 μg/mL puromycin (Yeasen). Western blotting was performed to confirm the efficacy of transfection. Supplementary Table 2 provides a comprehensive list of all targeted shRNAs.

### Assays of lipid metabolism

To quantify lipid droplets in cells, Nile red staining was performed. pNENs cells were transfected and seeded in 96-well plates. Once the cells reached 60-80% confluency, they were fixed in 4% paraformaldehyde for 15 minutes. Subsequently, the cells were incubated with Nile Red working fluid for 20 minutes, followed by staining with DAPI (Beyotime, Nantong, China) for 20 minutes at room temperature. Fluorescence intensity imaging of Nile Red and DAPI was acquired using fluorescence microscopy, and the fluorescence intensities were quantified using ImageJ software. The quantification of fatty acids (FAs) was performed using a CheKine™ Micro Free Fat Acid (FFA) Assay Kit (abbkine). Triglyceride and cholesterol contents were measured using EnzyChrom triglyceride and cholesterol kits (Bioassay Systems). All assays were conducted according to the manufacturer's instructions.

### Quantitative real time-polymerase chain reaction (qRT-PCR) and RNA-seq

Total RNA was isolated from cells using the trizol reagent (Vazyme, Nanjing, China) according to the manufacturer's protocol and quantified with a Nanodrop 2000. 5 µg of RNA was reverse transcribed to cDNA with the PrimeScript RT Reagent Kit with gDNA Eraser (Yeasen, Shanghai, China). Subsequently, real-time PCR was conducted using ChamQ Universal SYBR qPCR Master Mix according to the manufacturer's instructions. RNA samples for RNA-seq were sequenced by Lianchuan Biotech (Hangzhou, China) and analyzed using the OmicStudio tools available at https://www.omicstudio.cn/tool. The primers used are detailed in Supplementary Table 1.

### Protein extraction and western blot analysis

Total cellular proteins were lysed using cold NP40 lysis buffer (Beyotime, China) supplemented with protease inhibitors (Roche, Mannheim, Germany). The protein concentration was quantified using a BCA Protein Assay Kit (Beyotime, China). Subsequently, the samples were separated by 10% SDS-PAGE and transferred onto nitrocellulose membranes, which were then blocked with a blocking buffer. The nitrocellulose membranes were incubated with the appropriate primary antibody overnight, followed by incubation with peroxidase (HRP)-conjugated secondary antibodies for 1 hour. After three washes with TBST, the signals were detected using a chemiluminescence system (Bio-Rad, USA).

### Immunofluorescence

Cells were cultured in Confocal dish overnight to achieve a confluency of 60% to 80%. Subsequently, the cells were washed three times with PBS and fixed with 4% paraformaldehyde for 15 minutes. Following that, the cells were incubated with 0.2% Triton X-100 for 15 minutes and blocked with 3% BSA for 60 minutes. After three washes with PBST, the cells were incubated overnight at 4 °C with primary antibodies. The cells were then washed three times with PBST and incubated at room temperature for one hour with secondary antibodies. After three washes with PBST, the cells were incubated with DAPI for 15minutes. The fluorescence intensity was visualized using fluorescence microscopy.

### Quantification of global N6-methyladenosine levels

To assess the global level of RNA N6-methyladenosine (m6A), we employed the EpiQuik m6A RNA Methylation Kit (Epigentek, USA) in our study. Initially, total RNA was extracted from cells or tissues using TRIzol reagent. The RNA was then immobilized onto strip wells using the RNA high-binding solution. Subsequently, capture and detection antibodies were added sequentially to each well. Finally, the relative m6A level was determined by measuring the absorbance at a wavelength of 450 nm.

### M6a dot assay

Total RNAs from different group were initially denatured by heating at 65 °C for 5 minutes and then transferred onto a nitrocellulose membrane using a Bio-Dot apparatus (Bio-Rad, USA). Subsequently, the membranes were UV cross-linked, blocked, incubated overnight at 4 °C with m6A antibody (Abcam, USA). Following this, the membranes were incubated with peroxidase (HRP)-conjugated secondary antibodies for 1 hour. After 3 washes with PBST, the signals were detected using a chemiluminescence system. To ensure consistency among different groups, a membrane stained with 0.02% methylene blue (MB) in 0.3 M sodium acetate (pH 5.2) was utilized.

### Methylated RNA immunoprecipitation sequencing (MeRIP-seq)

The target gene was selected by MeRIP using the MeRIP m6A Kit (Merck Millipore) following the provider's requirements. Total RNA was extracted from *FTO* knockdown or empty vector QGP-1 cells using Seq-Star Poly(A) mRNA Isolation Kit. Next, the RNA was fragmented and incubated with m6A antibody to deposit *APOE*. After the concentration of m6A mRNA fragment and construction of the RNA-seq library for sequencing on the Illumina HiSeq 4000 platform, the abundance of *FABP5* was tested by qRT-PCR and normalized to the input mRNAs.

### Luciferase reporter assays

The wild-type (wt) and mutant (mut) 3′-UTR of *APOE* were constructed by RiboBio company (Guangzhou, China). Subsequently, different groups of cells were transfected with the luciferase constructs following the manufacturer's protocol. After 48 hours of transfection, luciferase activities were measured using a Dual-Luciferase Reporter Assay Kit (Promega). Each experiment was performed in triplicate.

### RNA immunoprecipitation (RIP) assays

The Magna RIP Kit (17-700, Millipore, MA) was used to perform the RNA immunoprecipitation (RIP) assay according to the manufacturer's instructions. In brief, 5 μg of specific antibodies against *IGF2BP2*, N6-methyladenosine (*m6A*), and anti-rabbit IgG were incubated with 40 μL magnetic beads. Then, cell lysates containing approximately 2 × 10^7^ cells per sample were added to the antibody-bead mixture. The RNA-protein IP complexes were washed six times to remove any non-specific binding. The RNAs of interest were then extracted and purified from the immunoprecipitated complex for further analysis using quantitative reverse transcription PCR (qRT-PCR). The relative enrichment of the target RNAs was normalized using the input RNA as a control.

### CO-IP assays

Briefly, beads were prewashed two times using 1× modified coupling buffer. Subsequently, they were incubated with 5 μg of the specified antibody for a duration of 15 min. Following this, the beads underwent washed three times using 1× modified coupling buffer, and the antibody was crosslinked using 1 ml of disuccinimidyl suberate solution for 30 min. The beads were then subjected to three washes using elution buffer, followed by two washes using IP lysis/wash buffer. The cell lysates were subsequently incubated with the beads for 8 h at 4 °C. On the following day, the beads were washed twice using 1 ml of IP lysis/wash buffer, and finally, a wash with ultrapure water was performed. The bound antigen was eluted and prepared for analysis via western blotting.

### FISH assays

Fluorescence *in situ* hybridization (FISH) assays were conducted using the *APOE* FISH Probe Mix Kit (RiboBio, Guangzhou, China). In brief, cells were fixed using 4% paraformaldehyde and subsequently treated with proteinase K and permeabilization solution. Prior to hybridization, pre-hybridization was performed at a temperature of 37 °C for a duration of 30 minutes. Subsequently, cells were subjected to hybridization with the fluorescently labeled APOE probe at a temperature of 37 °C overnight. Nuclei were counterstained with DAPI and visualized using a confocal laser microscope.

### Mouse xenograft model

A total of 5 × 10^6^ stably transfected QGP-1 cells were subcutaneously injected into the right axilla of female BALB/c nude mice (4-6 weeks old). After a period of 4-5 weeks, the mice were euthanized. Tumor formation and growth were monitored until the experimental endpoint, and tumor volume was calculated using the formula: (width)^2^ × length/2. The tumors were then either fixed in 4% paraformaldehyde or frozen for further analysis. All animal experiments conducted in this study were approved by the Institutional Animal Care and Use Committee (IACUC) of Nanjing Medical University.

### Statistical analysis

Statistical analysis was conducted using GraphPad Prism 8.0 software (GraphPad, Inc., USA). Student's t-test and one-way ANOVA with Tukey's multiple comparisons was employed to compare different groups. All experiments were repeated independently for a minimum of three times, and the results were expressed as means ± standard deviation (SD). The significance of the results was indicated on the figures, with a P-value less than 0.05 (P < 0.05) considered as statistically significant.

## Results

### *FTO* is overexpressed in pancreatic neuroendocrine neoplasms

**Figure 1:**
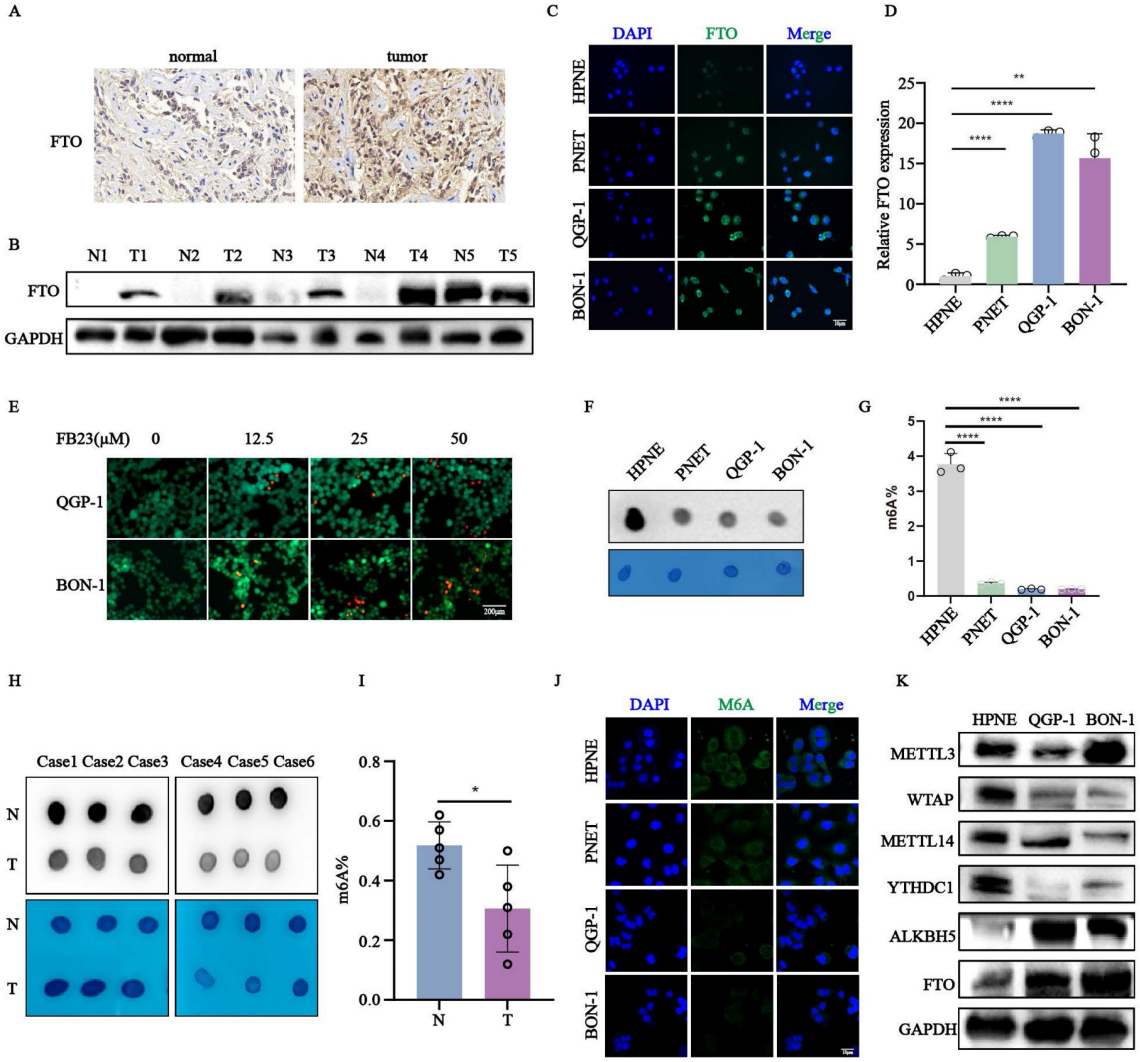
Increased expression of *FTO* was observed in pNENs. (A) Representative images of *FTO* immunohistochemistry staining in pNENs samples were shown. (B) The protein expression of *FTO* from five pairs of pNENs samples was indicated by western blots (N represents normal tissue, T represents tumor tissue). (C, D) The expression of *FTO* in pNENs cells (PNET, QGP-1, BON-1) compared to normal pancreatic cells HPNE verified by Immunofluorescence and qRT-PCR. (E) The cell mortality in different concentrations(µM) of cells treated with *FTO* inhibitor FB23 showed by Calcein AM/PI fluorescein staining. (F) M6A dot assays were performed to examine the amount of m6A in pNEN cells compared to normal pancreatic cells. (G) Global m6A levels in mRNA of pNENs cells and normal pancreatic cells were detected by an m^6^A quantification kit. (H) M6A dot assays in six pairs of pNENs samples were shown. (I) The percentage of m6A was examined in five pairs of pNENs samples. (J) The expression of m6A in pNENs cells compared to the normal pancreatic cell HPNE was verified by immunofluorescence. (K) The protein expression of m6A writers (*METTL3, METTL14, WTAP*), erasers (*FTO*, *ALKBH5*), and reader (*YTHDC1*) in pNENs cells (QGP-1, BON-1) compared to normal pancreatic cells HPNE was verified by western blots. *p < 0.01, **p < 0.01, ****p < 0.0001.

To investigate the role of *FTO* in pNENs, we first analyzed its expression in tumor and adjacent normal tissues using immunohistochemistry (Fig. 1A). *FTO* expression was elevated in most tumor samples compared to the corresponding normal adjacent tissues in five paired pNEN samples (Fig. 1B). Consistent with these tissue results, qRT-PCR and immunofluorescence assays showed elevated *FTO* mRNA and protein expression in pNENs cell lines (PNET, QGP-1, BON-1) compared to normal pancreatic cells (HPNE) (Fig. 1C-D). The treatment of pNENs cells with increasing concentrations(µM) of the FTO inhibitor FB23 resulted in a dose-dependent increase in cell mortality, indicating its anti-cancer effects (Fig. 1E, S1A-B). To determine whether the drug is targeted or toxic, we also treated with FB23 in HPNE cell, which began to exhibit toxic effects in HPNE cells at 100 µM (Fig. S1D). Additionally, global m6A levels were examined by m6A dots experiment in six pairs pNENs samples, revealing significantly lower m6A levels in tumors compared to normal pancreatic cells (Fig. 1H-I). Similar results were observed in pNENs cell lines compared to normal pancreatic cells (Fig. 1F-G,1J). We further elevated the protein expression of m6A regulators, including* METTL3, WTAP, METTL14,* and *YTHDC1, ALKBH5*, *FTO*, confirming the role of the m6A modification in pNENs progression (Fig. 1K). Moreover, the cell mortality was increased as the concentration(µM) of m6A nucleoside increased, indicating the an-cancer effect of m6A modification (Fig. S1C). In addition, we also excluded the influence the of drug toxicity which began to exhibit toxic effects in HPNE cells at 75 µM (Fig. S1D). Collectively, these results highlight the possible indispensable role of *FTO*-medicated m6A modification in pNENs development.

### *FTO* knockdown inhibits the malignant behavior of pNENs

**Figure 2:**
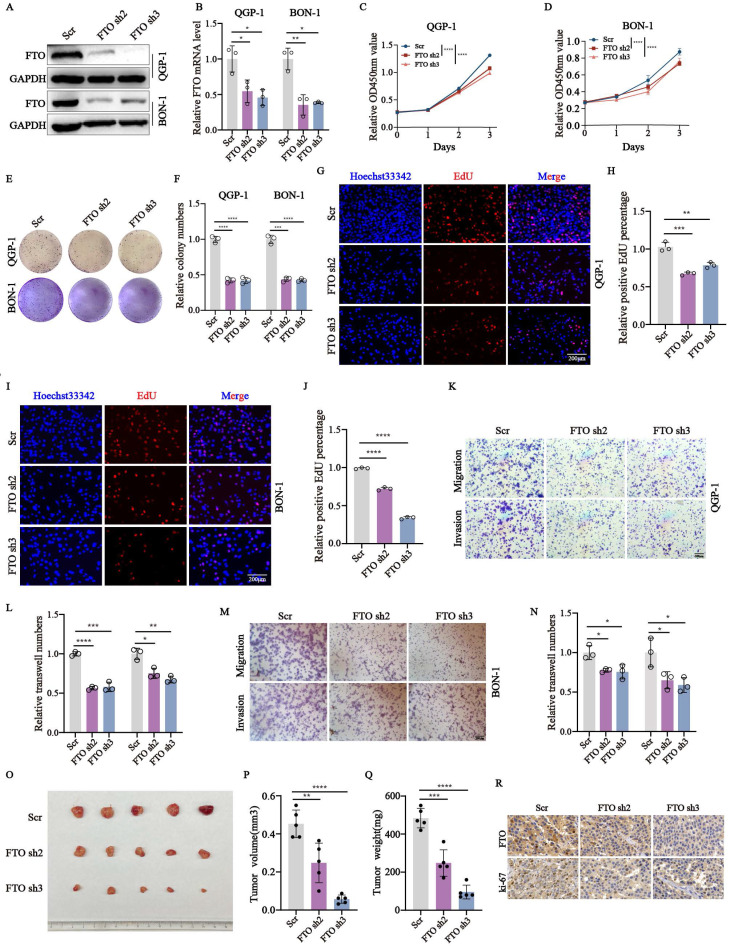
*FTO* knockdown repressed the proliferation, migration, and invasion of pNENs *in vitro* and *in vivo*. (A, B) The efficiency of *FTO* knockdown in QGP-1 and BON-1 cells was verified by qRT-PCR and western blots (Scr represents scramble which means control group with disrupted RNA sequence; Oc represents overexpression control). (C, D) CCK-8 assays were performed to elevate the proliferation rate of cells with *FTO* knockdown. (E, F) Colony formation assays were conducted in *FTO* deficiency cells. (G-J) EdU assays were performed to elevate the replication ability in *FTO* deficiency cells. (K-N) Transwell assays were carried out to examine the effects of *FTO* knockdown on pNENs cell migration and invasion. (O) Subcutaneous xenograft tumors derived from QGP-1 cells with *FTO* knockdown and the control group. (P-Q) Tumor volume and tumor weight between *FTO*-deficient groups and scramble groups. (R) Representative IHC staining images of FTO and ki-67. *p < 0.01, **p < 0.01, ***p < 0.001, ****p < 0.0001.

Since *FTO* has been demonstrated the crucial role plays in the malignant behavior of diverse cancers, we wondered whether *FTO* is involved in pNENs progression. To elucidate the biological role of *FTO* in pNENs, *FTO* knockdown stable cell lines were s. The efficiency of *FTO* knockdown was confirmed by q-RT-PCR and western blots (Fig. 2A-B). Next, we explored the proliferation rate of *FTO* knockdown in pNENs. CCK-8 and colony formation assays all indicated that the depletion of *FTO* markedly inhibited cell proliferation in pNENs cells (Fig. 2C-F). The result of the EdU assay also showed that *FTO* knockdown suppressed DNA replication activity in pNENs cells (Fig. 2G-J). Furthermore, transwell assays revealed that *FTO* knockdown inhibited migration and invasion in pNENs cells, as indicated by reduced staining of cells at the bottom of the chamber (Fig. 2K-N). Furthermore, we also established stable *FTO* overexpression cell lines, which exhibited notably up-regulated *FTO* mRNA and protein levels in QGP-1 and BON-1 cell lines (Fig. S2A-B). Conversely, ectopic *FTO* enhanced pNENs proliferation, as demonstrated by CCK8, colony formation, and EdU assay (Fig. S2C-H). Transwell assays also showed increased migration and invasion capacity in pNENs cells with *FTO* overexpression (Fig. S2I-L). To further investigate the role of *FTO* in pNENs progression *in vivo*, xenograft tumor experiments were performed by subcutaneously injecting QGP-1 cells into nude mice. The results indicated that *FTO* knockdown evidently inhibited pNENs growth, as evidenced by the reduced tumor volume, tumor weight, and immunohistochemical (IHC) staining of ki67 (Fig. 1O-R). Collectively, these data suggest that *FTO* knockdown inhibits pNENs progression.

### *FTO* regulates lipid metabolism pathways

**Figure 3:**
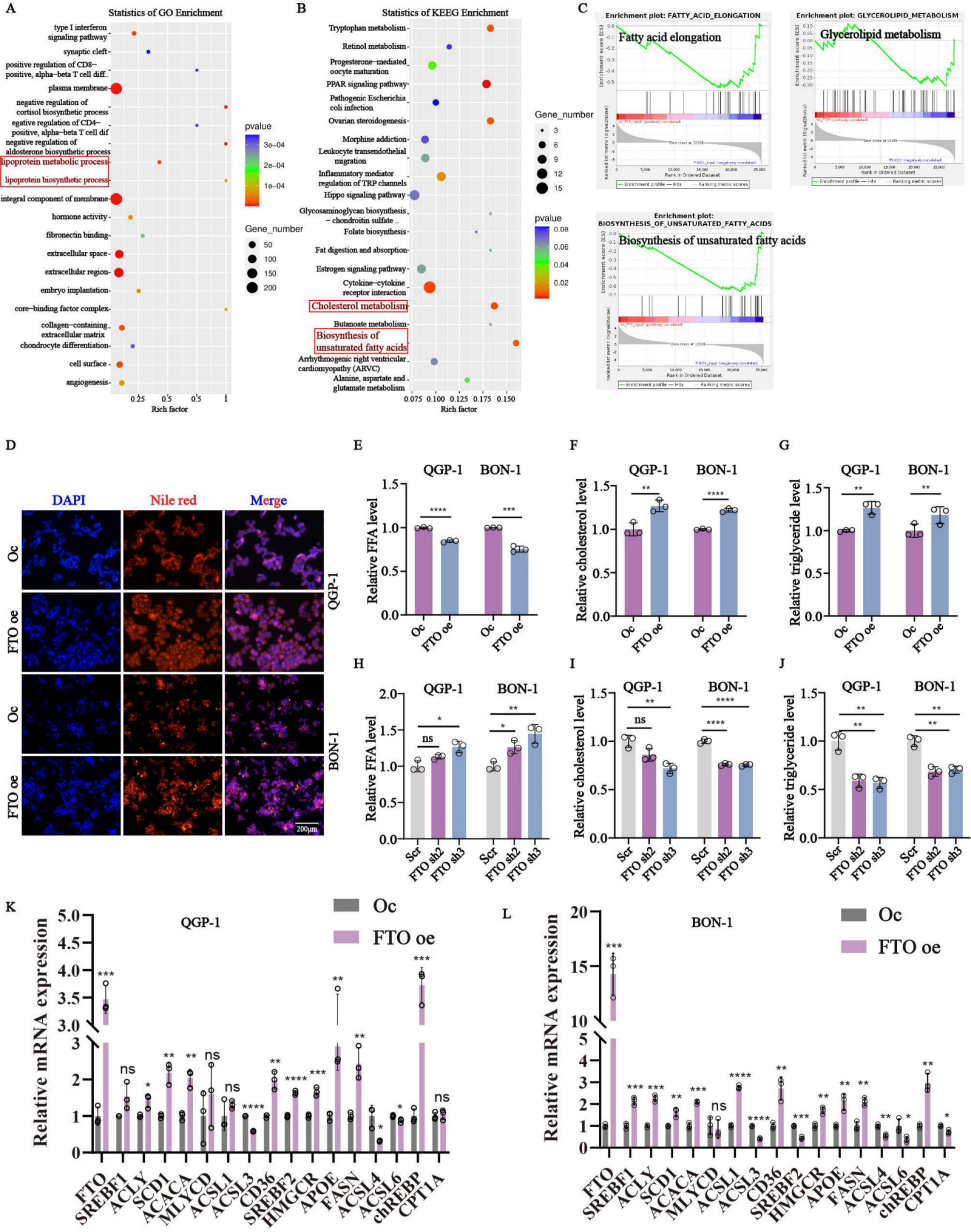
*FTO* knockdown impaired lipid metabolism of pNENs. Enrichment analysis of (A) GO and (B) KEGG signal pathways. (C) Individual GSEA plots of fatty acid elongation, glycerolipid metabolism, and biosynthesis of unsaturated acids pathway in RNA-seq data from QGP-1 cells with *FTO* knockdown. (D) LDs (lipid droplets), (E, H) FFA (free fatty acid), (F, I) cholesterol, and (G, J) triglyceride were examined in cells with *FTO* knockdown and *FTO* overexpression. (K, L) The relative mRNA expression of lipid-associated genes in cells with *FTO* overexpression. *p < 0.01, **p < 0.01, ***p < 0.001, ****p < 0.0001.

To better characterize how *FTO* promoted pNENs progression, RNA-seq was performed in *FTO* knockdown and control cells. Interestingly, two lipid metabolism-related pathways (lipoprotein metabolic process and lipoprotein biosynthetic process, cholesterol metabolism, and biosynthesis of unsaturated fatty acids) were all identified by GO enrichment and KEGG analysis respectively (Fig. 3A-B). As above, lipid metabolism has emerged as an important target for anti-tumor strategies, and its role in pNENs influenced by *FTO* remains unexplored. Therefore, we focused on unraveling the specific mechanism of *FTO* on lipid metabolism in pNENs. GSEA analysis revealed that *FTO* knockdown downregulated pathways involved in fatty acid elongation, biosynthesis of unsaturated fatty acids, and glycerolipid metabolism (Fig. 3C). To further explore the interrelationship between *FTO* and lipid metabolism, we performed a series of experiments about lipid metabolism. FTO overexpression increased lipid droplet accumulation and reduced free fatty acid content, indicative of enhanced lipid storage and lipolysis (Fig. 3D-E). Total cholesterol and triglyceride levels were also elevated in FTO-overexpressing cells, while FTO knockdown exerted the opposite effects (Fig. 3F-J). Moreover, abnormal lipid metabolism can result from enhanced lipid biosynthesis, decreased lipid catabolism, and increased fatty acid uptake. Further analysis revealed that FTO upregulated key lipid metabolism-related genes, including those involved in fatty acid synthesis (*SREBF1, ACLY, SCD1, ACACA, MLYCD, ACSL1, ACSL3, ACLS4, ACSL6, FASN, chREBP*), fatty acid uptake (*CD36*), fatty acid oxidation (*CPT1A*), cholesterol biosynthesis (*SREBF2, HMGCR, APOE*) (Fig. 3K,3L). It revealed the majority of molecules related to fatty acid synthesis, fatty acid uptake, and cholesterol biosynthesis are promoted by *FTO*. However, the molecules related to fatty acid oxidation seem not to be influenced by *FTO*. These findings underscore the pivotal role of FTO in lipid metabolism regulation in pNENs.

### *APOE* has been identified as the target gene regulated by *FTO*

**Figure 4:**
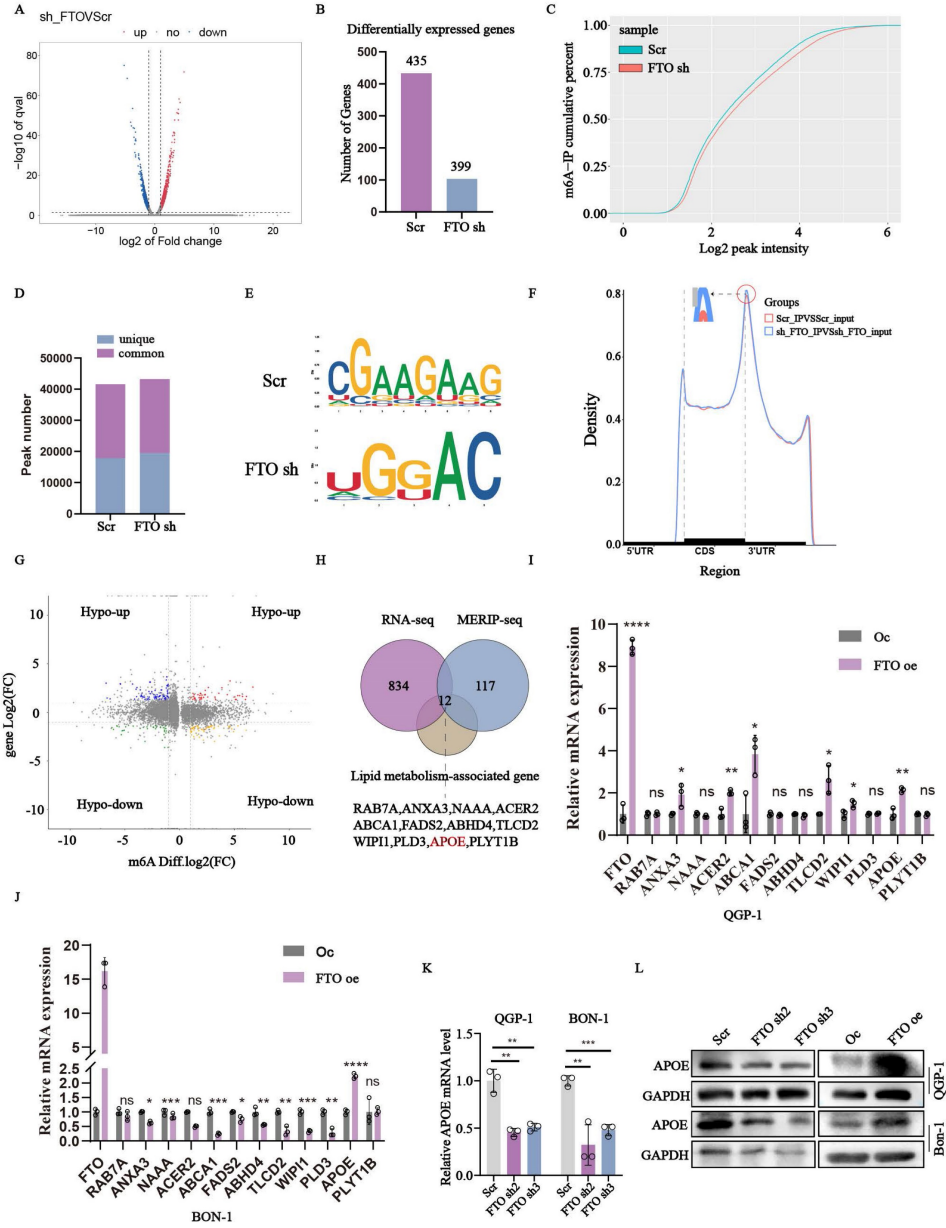
*APOE* was screened as the important target gene of *FTO*. (A, B) The differentially expressed genes in *FTO*-deficient QGP-1 cells compared to the control groups from the results of RNA-seq. (C) The m6A-IP cumulative percent between *FTO*-deficient QGP-1 cells compared to the control groups shown by the m6A saturation curve from the results of MeRIP-sequencing. (D) The peak profiles in m6A modification in cells with *FTO* knockdown as shown by MeRIP-sequencing. (E) The most common m6A consensus motif in *FTO*-deficient groups and the control groups. (F) Distribution of m6A peaks in *FTO*-deficient groups and the control groups. (G) The volcano plot indicated the distribution of genes both differential (up or down) methylation level and differential (up or down) gene expression levels in *FTO*-deficient groups and the control groups. (H) Venn diagram showed overlap genes among the significant genes from RNA-seq, the up-regulated methylation levels genes from MeRIP-sequencing, and lipid-associated genes. (I, J) The mRNA expression of the overlap genes between *FTO* overexpression groups and the control groups. The (K) mRNA and (L) protein expression of *APOE* in cells with *FTO* knockdown and overexpression was examined by qRT-PCR and western blots. *p < 0.01, **p < 0.01, ***p < 0.001, ****p < 0.0001.

To further elucidate the precise mechanism of *FTO* in pNENs lipid metabolism, we employed an integrated approach combing transcriptome and epitranscriptome sequencing in stable *FTO* deficiency QGP-1 cells and corresponding control cells. Transcriptome sequencing revealed significant differences in 834 genes, with 435 genes up-regulated and 399 genes down-regulated, all with a P-value below 0.05 (Fig. 4A-B). The m6A saturation curve further indicated a lower m6A saturation rate in FTO deficient cells (Fig. 4C). MeRIP-seq analysis revealed 17837 unique m6A peaks in the control group, 19459 unique m6A peaks in the sh-*FTO* group, and 23754 shared m6A peaks in both groups (Fig. 4D). The “CGAAGAAG” and “UGGAC” sequence motif were verified to be highly enriched in m6A-immunoprecipitated RNAs of the control and *FTO* knockdown cells (Fig. 4E). Because FTO knockdown positively mediates m6A modification, we focused on the mRNA transcripts with these enhanced m6A peaks, which were found to be predominantly localized in the CDS and 3'UTR region (Fig. 4F). Subsequently, Methylated RNA immunoprecipitation (MeRIP) was combined with m6A-specific antibody followed by RNA sequencing to accurately identify downstream targets (Fig. 4G). By comparing the overlapping parts of RNA-seq and MERIP-seq between the control and *FTO* knockdown, we identified 393 genes with significant differences. Among these genes, we excluded 276 genes with down-regulated m6a levels that were inconsistent with high global m6A levels regulated by *FTO* knockdown. Ultimately, the remaining twelve genes involved in lipid metabolism were singled out, including *RAB7A, ANXA3, NAAA, ACER2, ABCA1, FADS2, ABHD4, TLCD2, WIPI1, PLD3, APOE, PLYT1B* (Fig. 4H). To validate these findings, we assessed the mRNA levels of these candidate genes. qRT-PCR analysis revealed that *APOE* exhibited the most significant alteration in *FTO* over-expression cells (Fig. 4I-J). Additionally, we confirmed the expression of *APOE* in *FTO* knockdown cell lines through qRT-PCR (Fig. 4K). Correspondingly, the protein expression of APOE was also altered in FTO knockdown and overexpression cells (Fig. 4L). Collectively, these results indicated that *APOE* may serve as a target gene regulated by *FTO* in the context of PNENs lipid metabolism.

### Overexpression of *FTO* regulates *APOE* mRNA expression via an m6A-*IGF2BP2*-dependent mechanism

**Figure 5:**
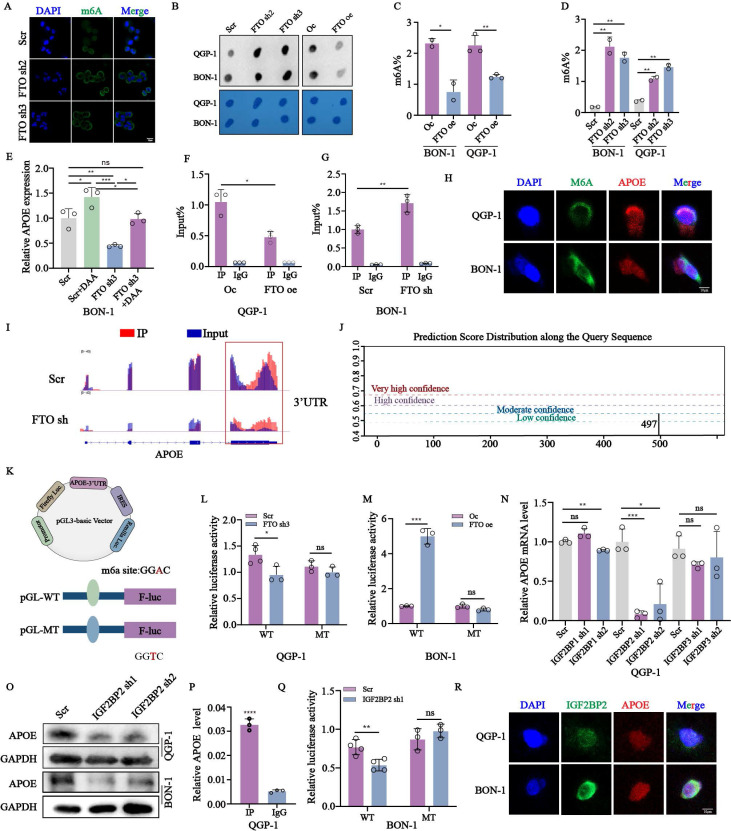
The expression of *APOE* was regulated by *FTO* in an m6A-*IGF2BP2* manner. (A)The expression of m6A in QGP-1 cells with *FTO* knockdown verified by immunofluorescence. (B) M6A dot assays were performed to assay the expression of m6A in *FTO* knockdown and overexpression cells. (C, D) The amounts of m6A were detected in *FTO* knockdown and overexpression cells. (E) The relative mRNA expression of *APOE* in QGP-1 cells treated with DAA. (F, G) RIP assays between m6A and *APOE* were performed in cells with *FTO* knockdown and overexpression. (H) FISH/IF assay showed co-localization of *APOE* mRNA and m6A proteins in the cytoplasm of pNENs cells. (I) The relative abundance of m6A sites along *APOE* mRNA in QGP-1 cells between *FTO*-deficient groups and the control groups from the results of MeRIP-seq. (J) The m6A modification sites in mRNA *APOE* were predicted by the website http://www.cuilab.cn. (K-M) The indicated wild-type (WT) and mutant-type (MUT) of pGL3-based luciferase reporter plasmids were used to examine the transcriptional activity of the *APOE* gene promoter affected by the m6A. We mutated base sequences of *APOE* from GGAC to GGTC. The firefly luciferase activity of *APOE* promoter was detected in QGP-1 cells with *FTO* knockdown and BON-1 cells with *FTO* overexpression. (N) The mRNA expression of *APOE* in QGP-1 cells with *IGF2BP1* knockdown, *IGF2BP2* knockdown, and *IGF2BP3* knockdown was verified by qRT-PCR. (O) The protein expression of the *APOE* in cells with *IGF2BP2* knockdown. (P) RIP assay performed between *IGF2BP2* protein and *APOE* mRNA in QGP-1 cells. (Q) The firefly luciferase activity of *APOE* promoter was detected in QGP-1 cells with *IGF2BP2* knockdown. (R) FISH/IF assay showed co-localization of *APOE* mRNA and *IGF2BP2* protein in the cytoplasm of pNENs cells. *p < 0.01, **p < 0.01, ***p < 0.001, ****p < 0.0001.

To further insight into the direct mechanism that *FTO* targets *APOE* mRNA and whether it depends on the m6A catalytic activity. We first assess the m6A levels in *FTO*-deficient cells using immunofluorescence (Fig. 5A). M6A dot-blot assays and RNA methylation quantification confirmed increased m6A levels in *FTO*-deficient cells and reduced levels in FTO-overexpressing cells (Fig. 5B-D). Treatment with the global methylation inhibitor 3-deazaadenosine (DAA) further indicated an inverse relationship between m6A levels and *APOE* expression (Fig. 5E). RIP assays demonstrated reduced interactions between m6A and *APOE* in *FTO*-overexpression cells, with the opposite observed in *FTO*-deficient cells (Fig. 5F-G). FISH/IF assays indicated cytoplasmic localization of the m6A-*APOE* combination (Fig. 5H). Using IGV analysis and the SRAMP server, we identified an m6A peak in the *APOE* 3ʹ untranslated region (UTR) near the stop codon, characterized by a conserved RRACH motif (Fig. 5I-J). To validate this finding, we mutated the GGAC motif to GGTC motif in *APOE* mRNA. Subsequently, we accurately mapped the location of the m6A peak across the human RNA transcriptome to construct luciferase reporter gene plasmids. The wild-type or mutated 3'-UTR of *APOE* was inserted downstream of the CDS region of the luciferase to assess the biofunction of m6A peaks. Both plasmids contained the Renilla luciferase (R-luc) gene as an internal reference (Fig. 5K). The results of the dual-luciferase reporter gene assay demonstrated a significant decrease in fluorescence after transfection of nonmutant plasmids in the *FTO* knockdown QGP-1 cells. Furthermore, there was no significant difference after transfection of mutant plasmids in the *FTO* knockdown cells (Fig. 5L). Conversely, opposite results were observed in BON-1 cells with *FTO* overexpression (Fig. 5M). To further explore the impact of m6A readers involved in the *FTO*-regulated m6A modification on *APOE*, we employed soft prediction to identify the protein *IGF2BPs* family. *IGF2BP2* was identified as the m6A reader regulating *APOE*. Knockdown of *IGF2BP2* significantly reduced *APOE* mRNA and protein expression (Fig. 5N-O). RIP and luciferase assays confirmed the interaction between *IGF2BP2* and *APOE* mRNA (Fig. 5P-Q). Collectively, these findings demonstrate that *FTO* and *IGF2BP2* regulate *APOE* in an m6A-dependent manner.

### *APOE* restores the malignant behaviors of *FTO* in pNENs *in vitro* and *in vivo*

**Figure 6:**
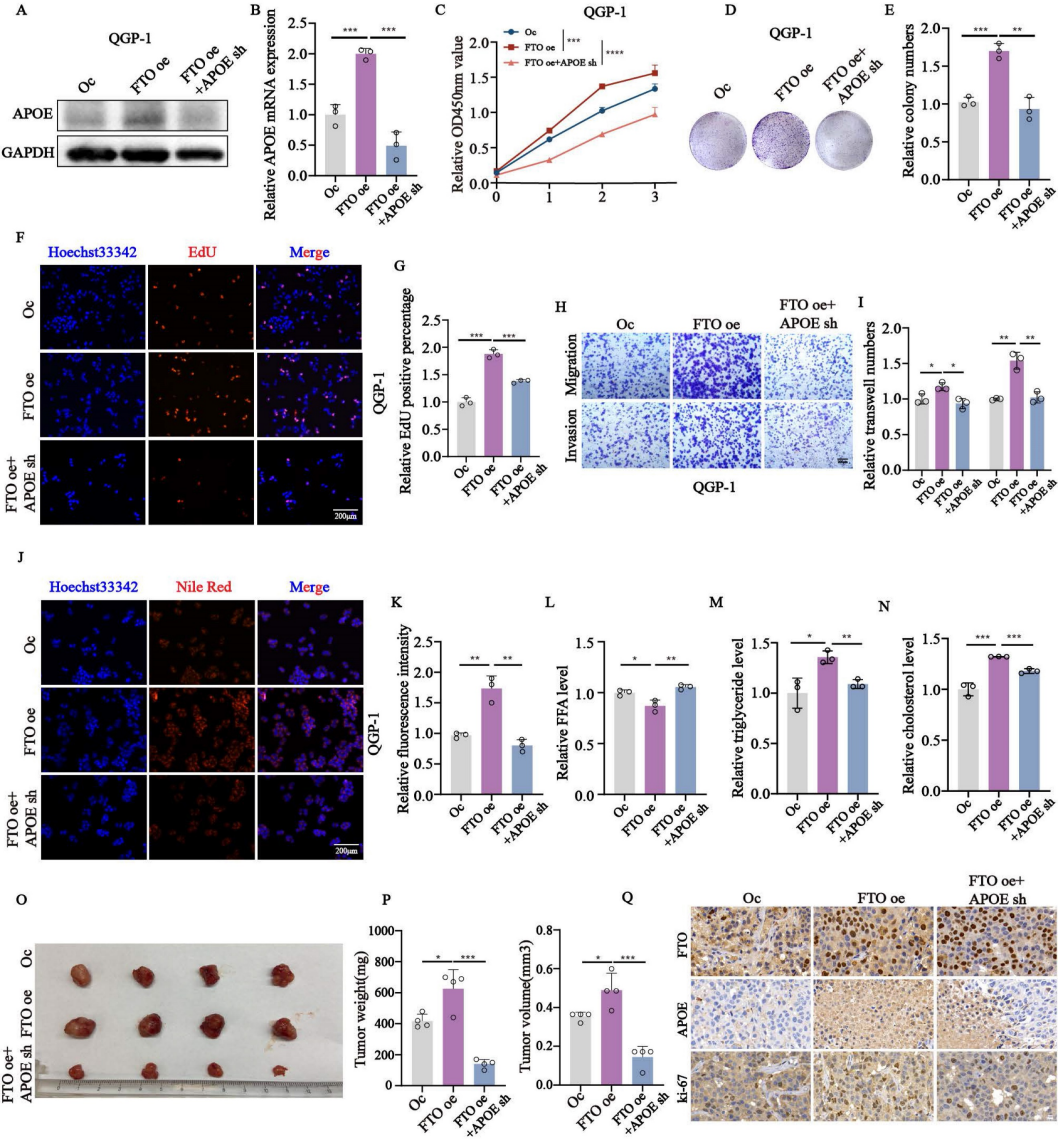
*APOE* knockdown partly reversed the effects of *FTO* overexpression on the proliferation and lipid metabolism of pNENs cells. (A, B) The mRNA and protein expression of *APOE* in the *FTO* overexpression cells with *APOE* knockdown. (C) CCK-8, (D, E) colony formation, and (F, G) EdU assay showed enhanced proliferation ability in QGP-1 cells with *FTO* overexpression was impaired by *APOE* knockdown. (H, I) Transwell assay indicated that *APOE* knockdown restored the ability of migration and invasion of *FTO* overexpression on QGP-1 cells. (J, K) Nile red assay showed that *APOE* knockdown reversed the formation of lipid droplets in cells with *FTO* overexpression. The relative levels of (L) FFA, (M) triglyceride, (N) cholesterol in *FTO* overexpression cells with *APOE* knockdown. (O-P) Representative images, tumor volume, and tumor weight of xenografts in the three groups. (Q) IHC staining of *FTO*, *APOE*, and ki-67 was performed in the xenografts of three groups. *p < 0.01, **p < 0.01, ***p < 0.001, ****p < 0.0001.

To evaluate the role of *APOE* in *FTO*-mediated tumorigenesis, we performed rescue assays *in vitro* and *in vivo* in *FTO* overexpression cell lines with or without *APOE* knockdown. Initially, we constructed the stable *FTO* overexpression with *APOE* knockdown cell lines using lentivirus verified by qPCR and western blots (Fig. 6A-B). *APOE* knockdown in *FTO*-overexpressing cells partially rescued proliferation and colony formation (Fig. 6C-E), as well as DNA replication (Fig. 6F-G). Knockdown of *APOE* also reversed *FTO*-induced migration and invasion capabilities (Fig. 6H-I). We also examined changes in the related index of lipid metabolism in *FTO* overexpression cells with or without *APOE* knockdown. The results showed that the knockdown of *APOE* reversed the accumulation of lipid droplets, as well as the levels of free fatty acids, triglycerides, and cholesterol influenced by *FTO* overexpression (Fig. 6J-N). Moreover, consistent with the *in vitro* results, *APOE* knockdown obviously weakened the tumor growth promoted by *FTO* overexpression, as reflected by tumor weight and tumor volume (Fig. 6O-P). Immunohistochemical staining of *APOE* and ki-67 in QGP-1 cells with *FTO* overexpression also showed rescue effects by *APOE* knockdown (Fig. 6Q). These findings suggest that *APOE* plays an important role in proliferation mediated by *FTO in vitro* and *in vivo*.

### *APOE* knockdown impairs the proliferation, migration, invasion, and lipid metabolism of pNENs cells

**Figure 7:**
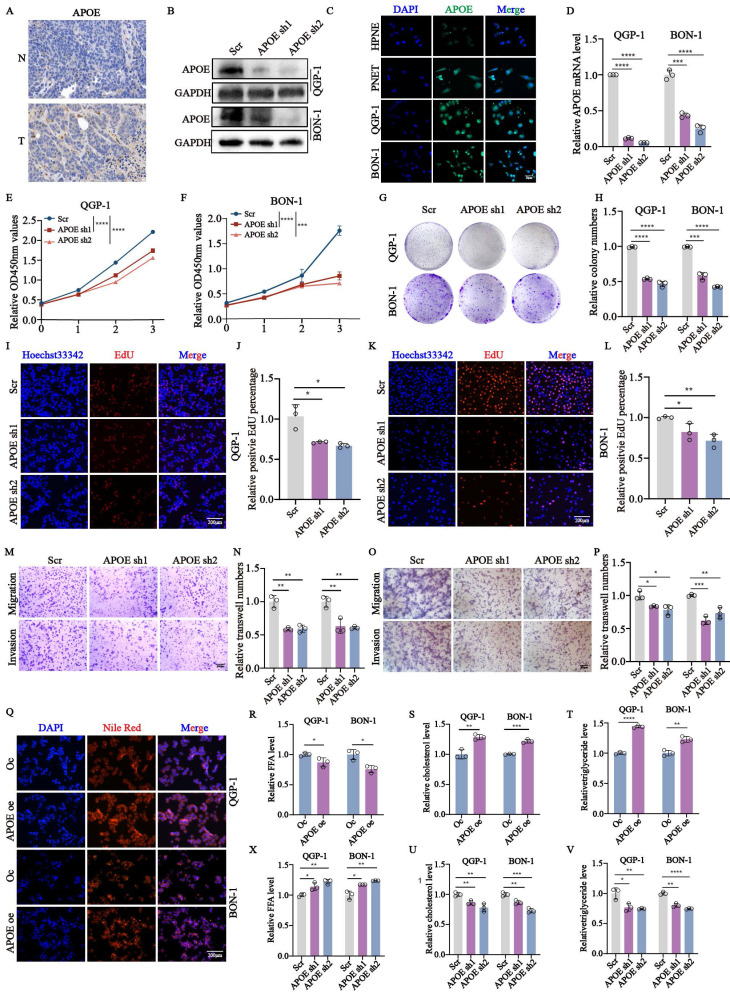
*APOE* knockdown inhibited the proliferation, migration, invasion, and lipid metabolism in pNENs. (A) Representative images of *APOE* immunohistochemistry staining in pNENs samples were shown. (B) The efficiency of *APOE* knockdown in pNENs cells was shown by western blots. (C) The expression of *APOE* in normal pancreatic cell line HPNE and pNENs cell lines PENT, QGP-1, and BON-1 was detected by immunofluorescent imaging. (D) The efficiency of *APOE* knockdown in pNENs cells was shown by qRT-PCR. The (E, F) CCK-8, (G, H) colony formation, (I-L) EdU assays were used to examine the proliferation ability of cells with *APOE* knockdown. (M-P) Transwell assays were applied to evaluate the ability of migration and invasion in *APOE* deficiency cells. (Q) LDs (lipid droplets), (R, X) FFA (free fatty acid), (S, U) total cholesterol, and (T, V) triglyceride were examined in cells with APOE overexpression and knockdown. *p < 0.01, **p < 0.01, ***p < 0.001, ****p < 0.0001.

To further investigate the biological functions of *APOE* in pNENs, we first examined the high expression of *APOE* in tumor tissues compared to normal tissues by using immunohistochemistry (Fig. 7A). The high expression of APOE was also found in pNENs cells (Fig. 7C). Furthermore, we also validated the mRNA and protein expression of *APOE* in cells with *APOE* knockdown (Fig. 7B,7D). Knockdown of *APOE* significantly reduced proliferation, as demonstrated by CCK-8, colony formation, and EdU assays (Fig. 7E-L). Transwell assays showed that APOE knockdown impaired migration and invasion (Fig. 7M-P). Moreover, we examined changes in the related index of lipid metabolism. The results showed that increased *APOE* expression led to higher total lipid droplet levels and lower free fatty acid levels (Fig. 7Q-R). The amounts of total cholesterol and triglycerides were also increased in cells with *APOE* overexpression (Fig. 7S-T). On the contrary, knockdown of *APOE* also had the opposite effects on pNENs in terms of the amount of free fatty acid, total cholesterol, and triglycerides (Fig. 7X-V). These results highlight APOE's pivotal role in pNEN progression.

### *APOE* protects *FASN* from the ubiquitin-proteasome degradation

**Figure 8:**
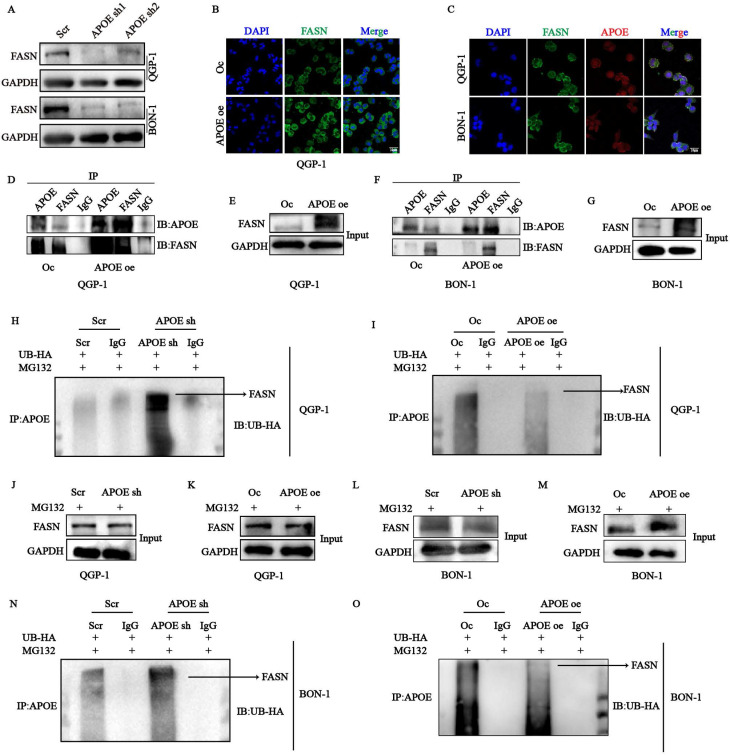
The stability of *FASN* was regulated by *APOE* in a ubiquitination-dependent way. (A) The protein expression of *FASN* in *APOE*-deficient cells was verified by western blots. (B) The expression of *FASN* in cells with *APOE* overexpression was verified by immunofluorescence (IF). (C) Immunofluorescence assay showed co-localization of *APOE* and *FASN* proteins in the cytoplasm of pNENs cells. (D-G) CO-IP assays between *APOE* and *FASN* protein were performed in cells with *APOE* overexpression. (H-K) QGP-1 cells with (H, J) *APOE* knockdown and (I, K) overexpression were transfected with UB-HA and then treated with MG132 (20µM) for 6h. Cell lysates were co-incubated with *APOE* and immunoblotting with the indicated antibodies. (L-O) BON-1 cells with (L, N) *APOE* knockdown and (M, O) overexpression were transfected with UB-HA and then treated with MG132 (20µM) for 6h. Cell lysates were co-incubated with *APOE* and immunoblotting with the indicated antibodies. *p < 0.01, **p < 0.01, ***p < 0.001, ****p < 0.0001.

To investigate the underlying regulatory mechanism of *APOE* in lipid metabolism, we performed mass spectrometry (MS) analysis to identify novel *APOE*-interacting proteins in QGP-1 cells with *APOE* overexpression. Among the crucial proteins involved in lipid metabolism, the existence of *FASN* caught our attention. We first confirmed the decreased protein expression of *FASN* in cells with *APOE* knockdown (Fig. 8A). The expression of *FASN* was also increased in cells with *APOE* overexpression verified by immunofluorescence (Fig. 8B). We further confirmed the interaction between *APOE* and *FASN* through confocal analysis, which showed colocalization of *APOE* and *FASN* in the cytoplasm of QGP-1 and BON-1 cells (Fig. 8C). CO-IP assay further demonstrated increased binding between *APOE* and *FASN* in cells with *APOE* overexpression compared to the control cells (Fig. 8D-G). To further elucidate the specific mechanism between *APOE* and *FASN*, the protein stability of *FASN* was examined in cells with *APOE* overexpression and knockdown. we first conjectured that *FASN* was regulated by the ubiquitin-proteasome system in the post-translational level. To further examine the ubiquitin-medicated regulation of *APOE* on *FASN*, we performed ubiquitination CO-IP. The results suggested that the ubiquitination level of *FASN* in cells with *APOE* knockdown increased compared with the level in control cells (Fig. 8H,8J,8N,8L), whereas the ubiquitination level of *FASN* in cells with *APOE* overexpression indicated the opposite results (Fig. 8I,8K,8O,8M). The overall data suggest that *FASN* is an *APOE*-interacting protein responsible for the regulation of *FASN* proteostasis.

### FB23 demonstrated anti-tumoral activity alone and in combination with everolimus in pNENs

To investigate the specific signal pathway involved in the development of pNENs. we singled out the *PI3K/AKT/mTOR* signal pathway for the results of KEGG analysis (Fig. S3A) and the application of the common drugs on pNENs, everolimus, a *mTOR* inhibitor.

**Figure 9:**
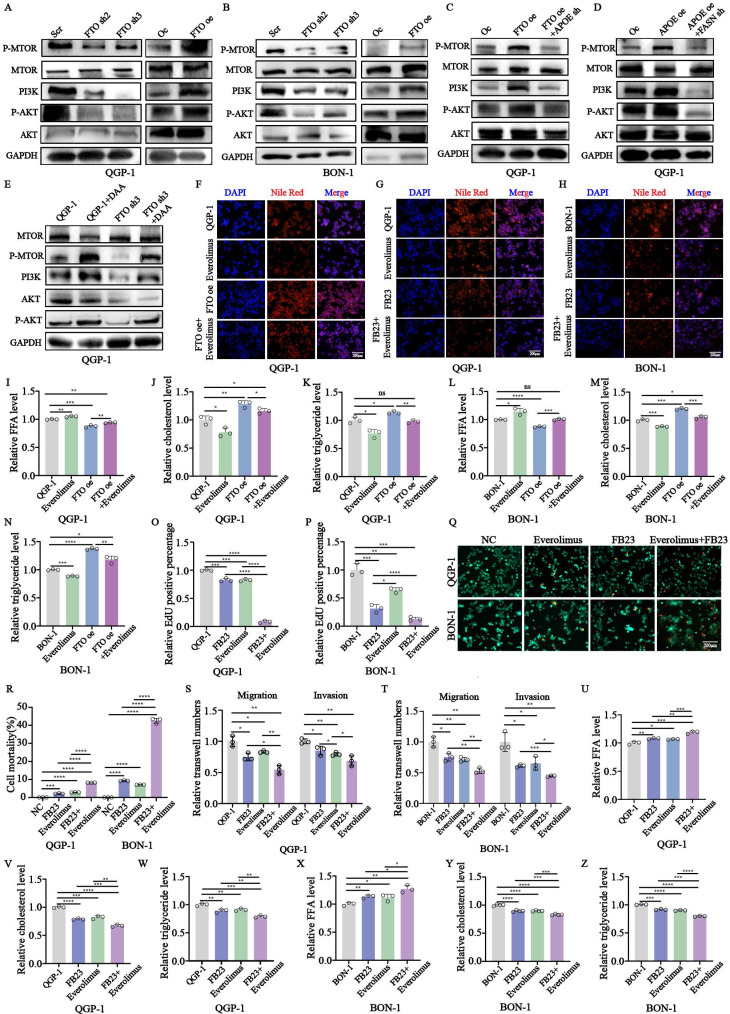
Evaluation of antitumoral activities of FB23-Everolimus combination in pNENs. (A, B) The related protein expression of *PI3K/Akt/mTOR* signaling pathway was performed by western blotting in pNENs cells with *FTO* knockdown and overexpression. (C) The related protein expression of *PI3K/Akt/mTOR* signaling pathway was performed by western blotting in *FTO* overexpression cells with or without *APOE* knockdown. (D) The related protein expression of *PI3K/Akt/mTOR* signaling pathway was performed by western blotting in *APOE* overexpression cells with or without *FASN* knockdown. (E) The related protein expression of *PI3K/Akt/mTOR* signaling pathway was performed by western blotting in *FTO* deficiency cells treated with DAA. (F) Lipid droplets were detected using Nile red in *FTO* overexpression QGP-1 cells treated with everolimus. (G, H) Lipid droplets were detected using Nile red in QGP-1 cells from the indicated groups. (I-N) The cellular content of free fatty acids (FFA), triglycerides, and total cholesterol were detected in QGP-1 cells from the indicated groups. (O-P) The results of EdU were conducted in cells from the indicated groups. (Q-R) Calcein AM/PI fluorescein staining was conducted in cells from the indicated groups. (S-T) Transwell assays were conducted in cells from the indicated groups. (U-Z) The cellular content of free fatty acids (FFA), triglycerides, and total cholesterol was detected in cells from the indicated groups. *p < 0.01, **p < 0.01, ***p < 0.001, ****p < 0.0001.

The results of western blots revealed that the crucial proteins involved in the *mTOR* signal pathway, *PI3K, P-AKT,* and *P-mTOR* were all upregulated in cells with *FTO* overexpression while knockdown of *FTO* had the opposite results (Fig. 9A-B). In addition, these enhancements could be rescued by *APOE* knockdown in cells with forced expression of *FTO* (Fig. 9C). The increase of these crucial proteins by *APOE* overexpression could also be reversed by *FASN* knockdown in cells with *APOE* overexpression (Fig. 9D). Moreover, we also further explore the influence of m6A modification on the *PI3K/Akt/mTOR* signaling pathway, using methylation inhibitor DAA. It indicated that the existence of DAA could partly rescue the inhibiting effect of *FTO* knockdown on the signaling pathway (Fig. 9E).

To further explore the interaction between *FTO* and lipid metabolism, the related index of lipid metabolism was also examined. The results showed that altered amounts of lipid droplets, cholesterols, and triglycerides could be rescued by the treatment of everolimus in cells with *FTO* overexpression (Fig. 9F,9I-N). Additionally, a significant decrease in cell proliferation, migration was induced by the combination compared to their respective FB23 and everolimus, showed by calcein AM/PI fluorescein staining, CCK-8, EdU assays, and transwell assays (Fig. 9Q-T; S3B-G).

However, we found the invasion of tumor do not indicate a significant change after the combination treatment (Fig. 9S-T; S3F-G). Moreover, the combination induced a more profound inhibitory effect of lipid metabolism than the respective FB23 and Everolimus (Fig. 9G-H, U-Z). Taken together, we found the *PI3K/Akt/mTOR* signaling pathway plays a crucial role in the development of pNENs and a combination between FB23 and everolimus led to more inhibition for the malignant role of pNENs which may provide an alternative therapeutic schedule for pNENs clinical therapeutics.

## Discussion

According to the previous study, low levels of m6A expression, associated with highly malignant behavior, have been observed in pNENs 25. In our study, we identified *FTO* as a highly upregulated m6A regulator in pNENs, suggesting its involvement in deregulated m6A modification driving oncogenic signaling. Notably, *FTO* exhibits carcinogenic effects by enhancing tumor proliferation, migration, and invasion during pNENs development. However, the role of m6A modification in cancer biology appears complex and contradictory. For instance, while *FTO* acts as an oncogene in pNENs, studies have demonstrated its anti-cancer role in other cancers. Silencing *FTO* inhibits cancer growth, cell motility, and invasion in hepatocellular carcinoma 26. Similarly, *FTO* overexpression enhanced chemoresistance in colorectal cancer by modulating SIVA1-mediated apoptosis 27. Beyond *FTO*, other m6A regulators, such as “writers”, also exhibit dual roles in cancer. For example, *METTL3* promotes tumor progression in colorectal and gastric cancers 28,29. Conversely, *WTAP* inhibits triple-negative breast cancer metastasis by downregulating *COL3A1* in an m6A-dependent manner 30. These discrepancies may stem from the heterogeneity of cell tissue samples, the distinct roles of m6A-related proteins, the different types of target genes (pro-cancer or anti-cancer), and other contributing factors 31. Regarding pNENs, the roles of m6A writers and readers remain largely unexplored. Further investigation into the complex interplay of these regulators is essential to fully understand the significance of m6A modifications in pNENs pathogenesis.

The metabolic adaptations of cancer cells, which enable uncontrolled proliferation, are critical in tumor development. Therefore, it is crucial to unravel the intricate metabolic adaptations that occur when cancer cells undergo this switch. Such understanding may pave the way for identifying pharmacological targets and potential diagnostic or prognostic biomarkers. For lipid metabolism, it is associated with many kinds of organ dysfunction and disease development, particularly in cancer cells. In the present study, we have discovered the presence of altered lipid metabolism, regulated by m6A modification, during the development of pNENs, corroborating previous findings 25. Moreover, mounting evidence suggests that modulating cell lipid metabolism could be an effective strategy for inhibiting cell proliferation in various cancers. For instance, *CDKN2A* deletion remodeled lipid metabolism in the development of prime glioblastoma 32. The NFYAv1-lipogenesis axis exerted tumor-promoting effects, indicating that lipid metabolism may be a potential therapeutic target for triple-negative breast cancer 33. In hepatocellular carcinoma, *SREBP1c* increased free fatty acids and promoted cell proliferation 34. Apart from lipid metabolism, other types of metabolic changes also play an indispensable role in tumors. For glycolysis in cancers, targeting ACYP1-mediated glycolysis could reverse lenvatinib resistance and restrict hepatocellular carcinoma progression 35. In glioblastoma, the mutation of *GLUT1* Cys207 to serine impaired glycolysis and cell proliferation 36. For amino acid metabolism in cancers, the transcription factor *ATF3* regulated serine and nucleotide metabolism to maintain cell cycling, survival, and the blockade of differentiation in acute myeloid leukemia 37. Phosphoglycerate dehydrogenase (*PHGDH*), the first rate-limiting enzyme of serine synthesis, was overexpressed and associated with the development of cancers 38. However, the role of these metabolic adaptations in pNENs still requires further exploration.

In most recent studies, *APOE* was regarded as a cancer-promoting factor. For example, *APOE* is elevated in prostate cancers and correlates with a poor prognosis 39. Conversely, *APOE* assumes an immunosuppressive role in the pancreatic cancer microenvironment by upregulating the expression of *CXCL1* and *CXCL5* 40. A similar immune suppression role of *APOE*, through binding the *LRP8* receptor on MDSCs, was also observed in melanoma 41. Furthermore, *APOE* could also lead to the abnormal cell proliferation of ovarian cancer by inhibiting cell cycle arrest and apoptosis 42. In our study, we found *APOE* could regulate lipid metabolism to further influence the pNENs progression. We revealed that the expression of *APOE* was dependent on an *FTO*-*IGF2BP2* manner. However, the specific underlying mechanisms of m6A-modified *APOE* in pNENs, such as RNA stability, nuclear export, and precursor RNA splicing, remain largely unknown. Moreover, to gain further insight into the specific underlying mechanism of *APOE* in lipid metabolism, we found that *APOE* enhanced lipid metabolism by regulating the ubiquitination-mediated expression of *FASN* in pNENs. However, the specific regulatory mechanism of *FASN* ubiquitination regulated by *APOE* has not been elucidated.

*APOE*, a secreted protein, has traditionally been recognized for its role in regulating lipid metabolism. However, its emerging significance in shaping tumor microenvironment has drawn increasing attention in recent years. Many studies have highlighted how *APOE* influences immune cell behavior and tumor-immune interactions, suggesting that it plays a critical role in modulating the immune microenvironment. This newfound understanding is reshaping perspectives on *APOE*, not only as a regulator of metabolic processes but also as a key player in tumor immunology. For instance, prostate tumor cells secrete *APOE*, which binds to *TREM2* on neutrophils to promote their senescence 39. *LXR/APOE* activation therapy has been explored as a potential target to enhance cancer immunotherapy by modulating innate immune suppression 41. Furthermore, *APOE* has also been identified as a prognostic biomarker correlated with immune infiltration in papillary thyroid carcinoma 43. Although *APOE* plays an essential role in the immune microenvironment of various tumors, its contribution to immune regulation in pNENs remains largely unexplored and warrants further investigation.

In summary, we reveal that overexpressed *FTO* is responsible for increased expression of *APOE* in an m6A-dependent manner, thereafter aggravating lipid accumulation by inhibiting the ubiquitination degradation of *FASN*, which further promotes the malignant progression of pNENs. Our study demonstrates a novel regulating mechanism for lipid metabolism via epitranscriptomics, which provides an alternative elucidation for pNENs pathogenesis.

## Supplementary Material

Supplementary figures and tables.

## Abbreviations

pNENs: pancreatic neuroendocrine neoplasms
m6A: N6-methyladenosine
*FTO*: Fat-mass and obesity-associated protein
*ALKBH5*: AlkB homolog 5
*METTL3*: Methyltransferase-like 3
*METTL14*: Methyltransferase-like 14
*WTAP*: Wilms tumor 1 associated protein
IGF2BP: Insulin-like growth factor 2 mRNA-binding proteins
MeRIP: Methylated RNA immunoprecipitation
RIP: RNA Immunoprecipitation
DAA: 3-Deazaadenosine
CCK8: Cell counting kit-8
EdU: 5-Ethynyl-2'-deoxyuridine
qRT-PCR: Quantitative real-time polymerase chain reaction
KEGG: Kyoto Encyclopedia of Genes and Genomes
GO: Gene ontology
IHC: Immunohistochemistry
GAPDH: Glyceraldehyde-3-phosphate dehydrogenase
