# *FTO*-induced *APOE* promotes the malignant progression of pancreatic neuroendocrine neoplasms through *FASN*-mediated lipid metabolism: Erratum

**DOI:** 10.7150/ijbs.132859

**Published:** 2026-03-27

**Authors:** Jinhao Chen, Mujie Ye, Danyang Gu, Ping Yu, Lin Xu, Bingyang Xue, Lijun Yan, Feiyu Lu, Chunhua Hu, Yanling Xu, Xiaoting Shi, Lingyi Chen, Yan Wang, Jianan Bai, Ye Tian, Qiyun Tang

**Affiliations:** 1 Department of Geriatric Gastroenterology, Neuroendocrine Tumor Center, Jiangsu Province Hospital, The First Affiliated of Nanjing Medical University, Institute of Neuroendocrine Tumor, Nanjing Medical University, Nanjing, China.; 2 Center for Biomarker Discovery and Validation, National Infrastructures for Translational Medicine (PUMCH), Institute of Clinical Medicine, Peking Union Medical College Hospital, Chinese Academy of Medical Science & Peking Union Medical College, Beijing, China.; 3 Digestive Endoscopy, Jiangsu Province Hospital, The First Affiliated Hospital of Nanjing Medical University, China.; 4 Department of Gastroenterology, The Friendship Hospital of Ili Kazakh Autonomous Prefecture, Ili State 835000, China.

In the published version of this paper, the author noticed a non-subjective error in Figure 2E. Specifically, the image of BON-1 cells transfected with FTO sh2 was inadvertently duplicated and incorrectly used to represent BON-1 cells transfected with FTO sh3.

We have carefully re-examined the original raw data and confirmed that this error does not affect the results or conclusions of the study. The correct image for the FTO sh3 group has been provided, and all authors have agreed to this correction. We sincerely apologize for any inconvenience caused by this oversight and remain committed to maintaining the accuracy and integrity of the scientific record.

Figure 2E should be corrected as follows.

## Figures and Tables

**Figure 2:**
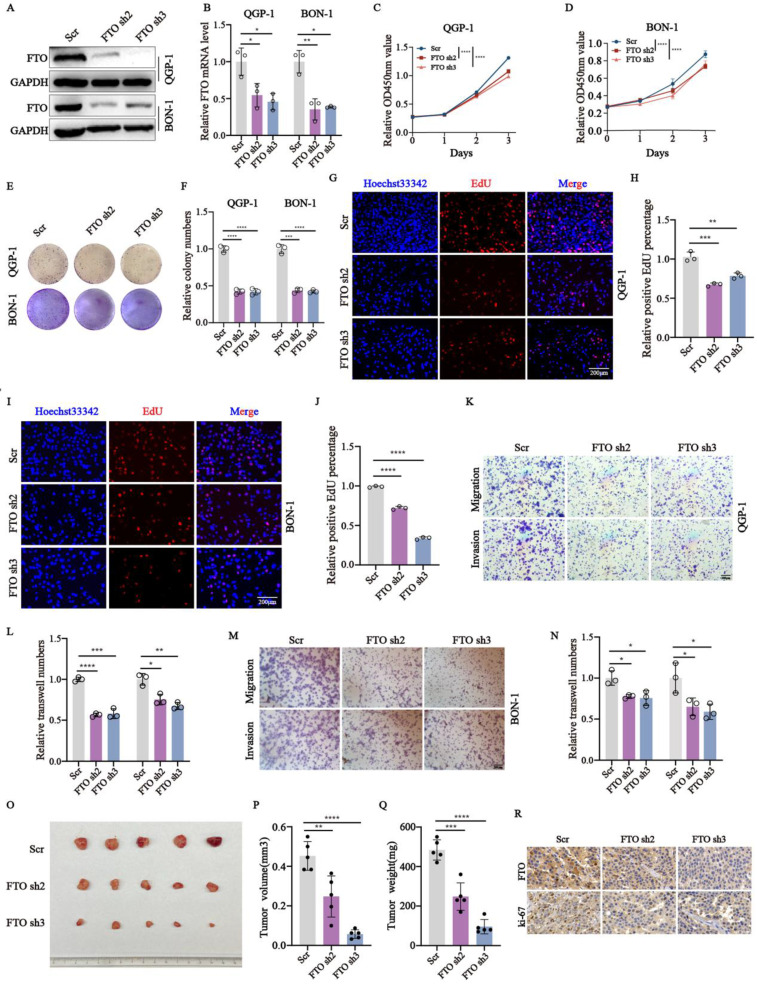
Correct image.

