# Supplementary material for: FTO-induced APOE promotes the malignant progression of pancreatic neuroendocrine neoplasms through FASN-mediated lipid metabolism
Source: Int J Biol Sci. 2025 Jan 27;21(4):1478–96. doi: 10.7150/ijbs.103428 (PMC11844274; doi:10.7150/ijbs.103428)

Supplementary Table1 Primers of genes

Gene names	Sequence(5'-3')
chREBP forward	AAGATCCGCCTGAACAACG
chREBP reverse	CACTTGTGGTATTCCCGCATC
FASN forward	AAGGACCTGTCTAGGTTTGATGC
FASN reverse	TGGCTTCATAGGTGACTTCCA
ACLY forward	TCGGCCAAGGCAATTTCAGAG
ACLY reverse	CGAGCATACTTGAACCGATTCT
ACSL4 forward	CATCCCTGGAGCAGATACTCT
ACSL4 reverse	TCACTTAGGATTTCCCTGGTCC
ACSL6 forward	GCACGGCGATCTGTGATTG
ACSL6 reverse	GATGTAGCGGATAGCCCCAG
GAPDH forward	GGAGCGAGATCCCTCCAAAAT
GAPDH reverse	GGCTGTTGTCATACTTCTCATGG
SREBF1 forward	ACAGTGACTTCCCTGGCCTAT
SREBF1 reverse	GCATGGACGGGTACATCTTCAA
CD36 forward	GGCTGTGACCGGAACTGTG
CD36 reverse	AGGTCTCCAACTGGCATTAGAA
CPT1A forward	TCCAGTTGGCTTATCGTGGTG
CPT1A reverse	TCCAGAGTCCGATTGATTTTTGC
ACSL1 forward	CCATGAGCTGTTCCGGTATTT
ACSL1 reverse	CCGAAGCCCATAAGCGTGTT
ACSL3 forward	GCCGAGTGGATGATAGCTGC
ACSL3 reverse	ATGGCTGGACCTCCTAGAGTG
SCD1 forward	TCTAGCTCCTATACCACCACCA
SCD1 reverse	TCGTCTCCAACTTATCTCCTCC
ACACA forward	ATGTCTGGCTTGCACCTAGTA
ACACA reverse	CCCCAAAGCGAGTAACAAATTCT
HMGCR forward	TGATTGACCTTTCCAGAGCAAG
HMGCR reverse	CTAAAATTGCCATTCCACGAGC
FADS2 forward	TGACCGCAAGGTTTACAACAT
FADS2 reverse	AGGCATCCGTTGCATCTTCTC
RAB7A forward	GTGTTGCTGAAGGTTATCATCCT
RAB7A reverse	GCTCCTATTGTGGCTTTGTACTG
ANXA3 forward	TTAGCCCATCAGTGGATGCTG
ANXA3 reverse	CTGTGCATTTGACCTCTCAGT
NAAA forward	CAACCTGGCCTACGAGTCC
NAAA reverse	GCTTGCGTAAGACATTCCCAAAA
ACER2 forward	TGGTGCGAGGACAACTACAC
ACER2 reverse	GCAGATGGGCGGTAAAATGAA
ABCA1 forward	ACCCACCCTATGAACAACATGA
ABCA1 reverse	GAGTCGGGTAACGGAAACAGG
ABHD4 forward	TCCCCTCCGACCAACTAACC
ABHD4 reverse	AGCTACTCGAAGAACAGCCAA

TLCD2 forward	CCTGTCACTGTACCCTCAGAT
TLCD2 reverse	TGCCAGGAAGTAACCCACAGA
WIPI1 forward	ACTAAAGCCGGGTATAAGCTGT
WIPI1 reverse	CGGGATTTCATTGCTTCCGTG
PLD3 forward	AAGCCTAAACTGATGTACCAGGA
PLD3 reverse	GCCTCAATCTCATTCATGGGC
PCYT1B forward	CCATGAAAAACTGACCATTGCT
PCYT1B reverse	GCATAAGGGCTCTTGCATGAC
FTO forward	GCTGCTTATTTCGGGACCTG
FTO reverse	AGCCTGGATTACCAATGAGGA
APOE forward	GTTGCTGGTCACATTCCTGG
APOE reverse	GCAGGTAATCCCAAAAGCGAC

Supplementary Table2 Short hairpin targets

Gene names	Target sequence(5'-3')
APOE sh1	GAAGGCCTACAAATCGGAACT
APOE sh2	GCAGACACTGTCTGAGCAGGT
FTO sh2	CGGTTCACAACCTCGGTTTAG
FTO sh3	ACCTGAACACCAGGCTCTTTA
IGF2BP1 sh1	GCAGTGGTGAATGTCACCTAT
IGF2BP1 sh2	CTCCAAAGTTCGTATGGTTAT
IGF2BP2 sh1	AGTGAAGCTGGAAGCGCATAT
IGF2BP2 sh2	CAGTGCTGAGATAGAGATTAT
IGF2BP3 sh1	GAAACTTCAGATACGAAATAT
IGF2BP3 sh2	TCTGCGGCTTGTAAGTCTATT
FASN sh1	CCTACTGGATGCGTTCTTCAA
FASN sh2	GCTACGACTACGGCCCTCATT

SupplementaryTable3 Antibody information

Antibody	Company	Catalogue	Dilution ratio
GAPDH	Proteintech	60004-1-Ig	1：5000
mTOR	CST	2983S	1：1000
p-mTOR2481	CST	2974S	1：1000
PI3K	Proteintech	20584-1-AP	1：1000
AKT	CST	9272S	1：1000
p-AKT ser473	CST	4060T	1：1000
APOE	Abcam	ab52607	1：1000
FASN	CST	3180S	1：1000
ALKBH5	Abcam	ab195377	1：1000
FTO	Abcam	ab126605	1：1000
Goat Anti-Rabbit IgG	CWBIO	CW0103S	1：2000
Goat Anti-Mouse IgG	CWBIO	CW0102S	1：5000
β-Tubulin	Proteintech	10068-1-AP	1：5000
Ki67	Proteintech	27309-1-AP	1 : 2000
METTL3	Abcam	ab195352	1：1000
METTL14	Abcam	ab300104	1：1000
WTAP	Abcam	ab195380	1：1000
IGF2BP2	Proteintech	11601-1-AP	1：1000
HA	Proteintech	51064-2-AP	1：1000

FigureS1 FB23 and m6A nucleoside inhibited cell mortality.

(A, B) The cell mortality in different concentrations(μM) of cells treated with FB23 showed by Calcein AM/PI fluorescein staining. (C) The cell mortality in different concentrations(μM) of cells treated with m6A nucleoside showed by Calcein AM/PI fluorescein staining. (D) Calcein AM/PI fluorescein staining was conducted in normal pancreatic cells HPNE treated with FB23 and m6A nucleoside.

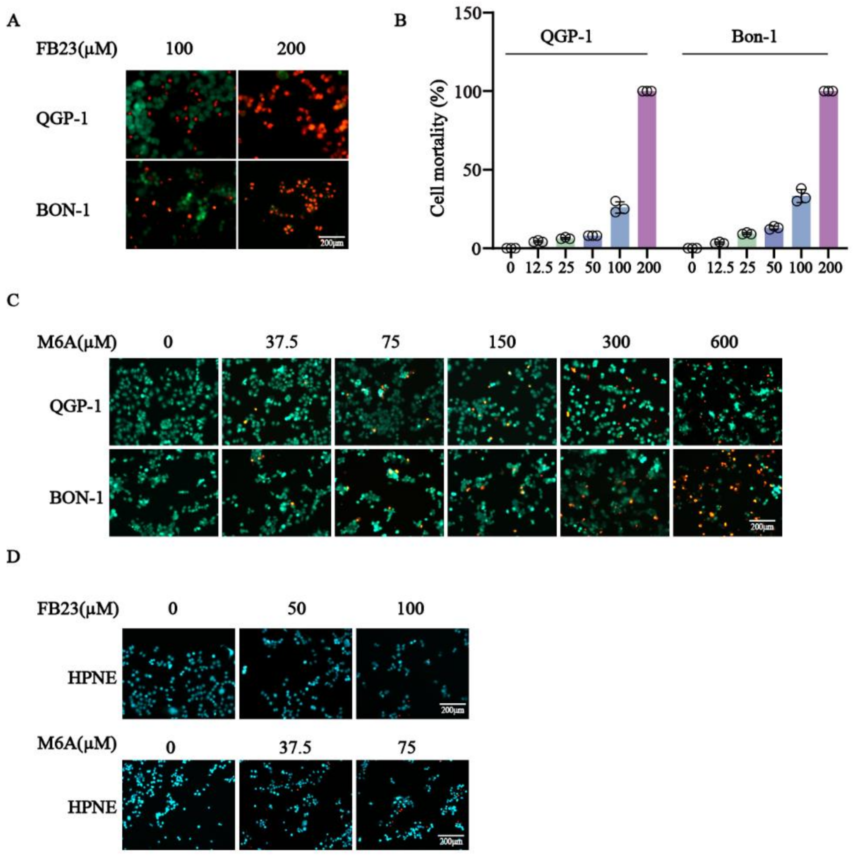

FigureS2 *FTO* overexpression promoted the proliferation, migration, and invasion in pNENs cells.

(A, B) The mRNA and protein expression of *FTO* in cells with *FTO* overexpression verified by qRT-PCR and western blots. (C-H) The results of CCK-8, colony formation, and EdU assays in cells with *FTO* overexpression. (I-L) The results of Tranwell assays in cells with *FTO* overexpression.

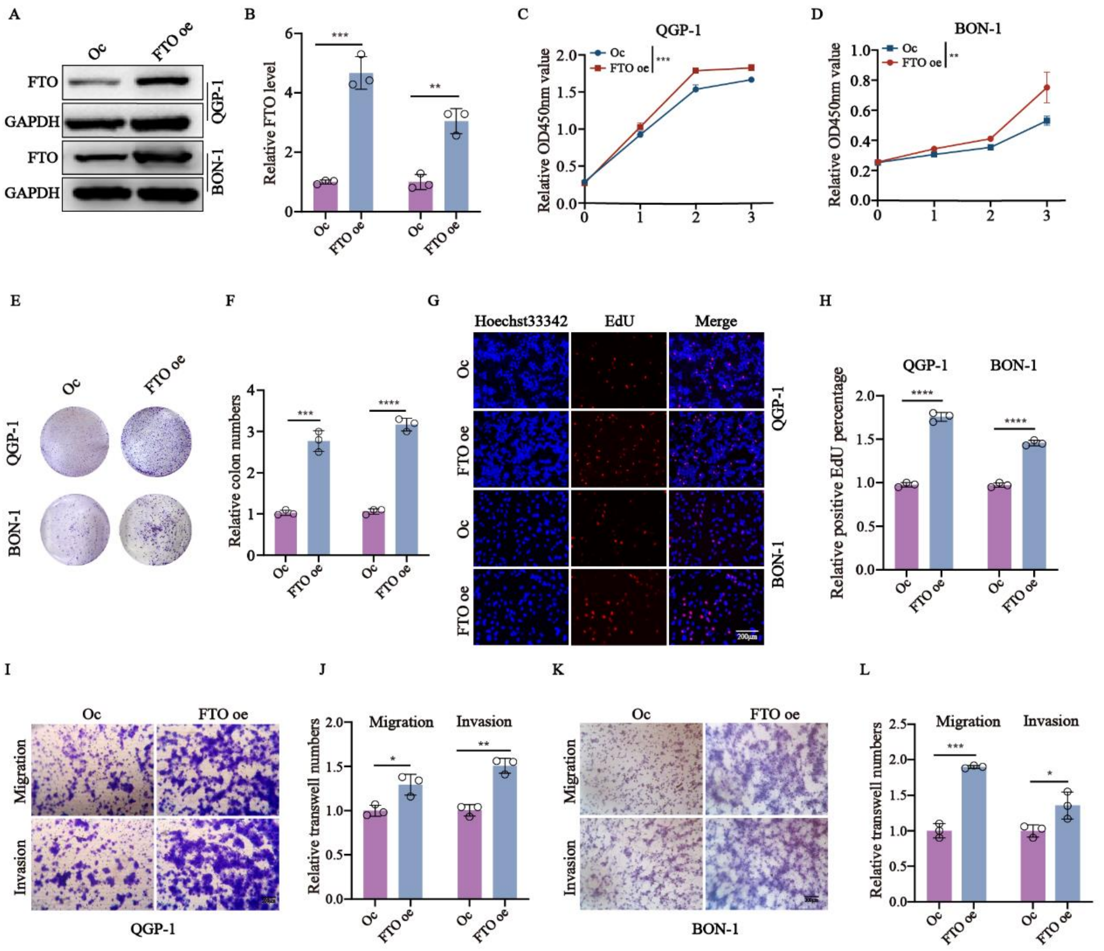

FigureS3 (A) Enrichment barplot showing the KEGG analysis of RNA-seq data derived from FTO knockdown and scramble control cells. (B, C) The results of CCK-8 in cells treated with FB23 and Everolimus. (D, E) The results of EdU in cells treated with FB23 and Everolimus. (F, G) The results of transwell assay in cells treated with FB23 and Everolimus.

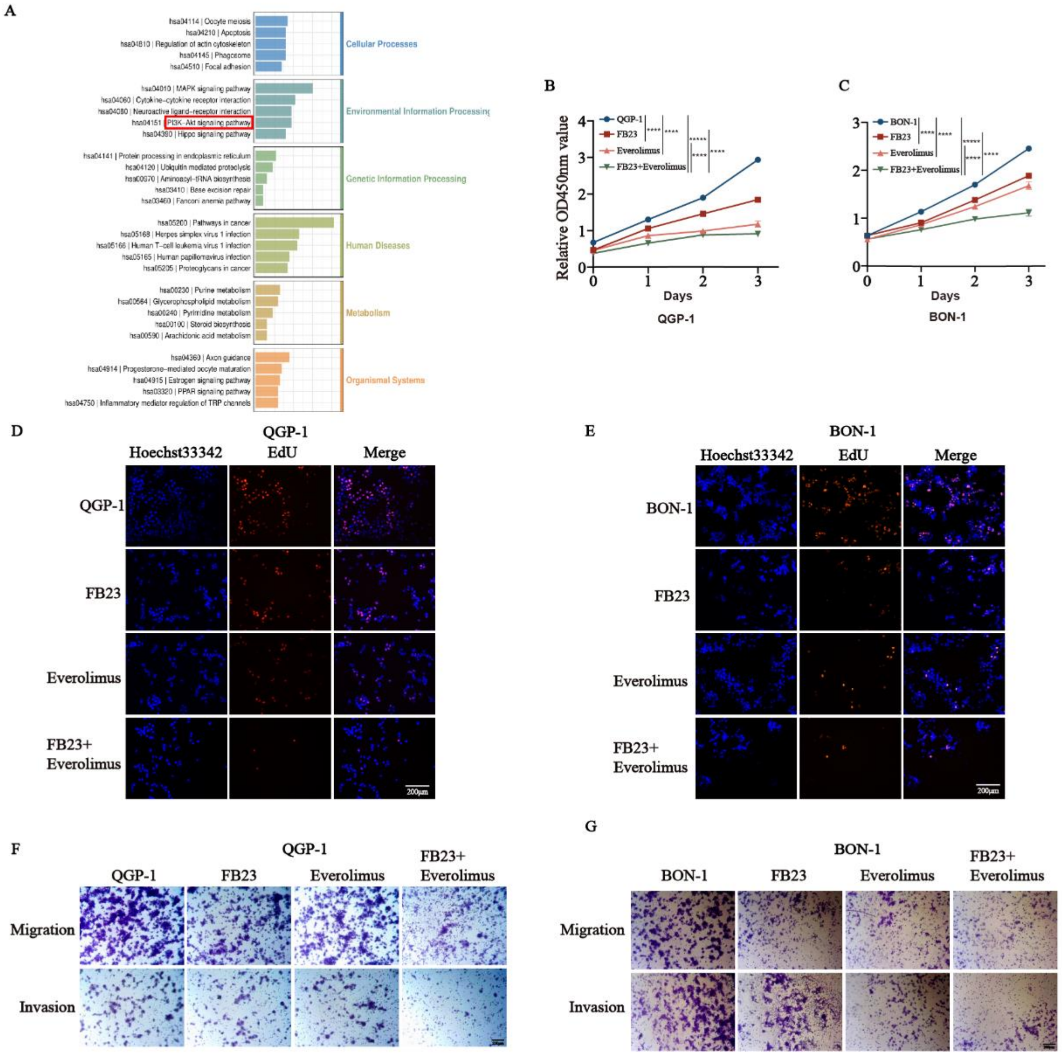

Supplement: Supplementary file 1 — Supplementary figures and tables. [file ijbsv21p1478s1.pdf]
